# Der Driburger Kreis – Eine Institution der deutschen Wissenschaftsgeschichte

**DOI:** 10.1007/s00048-024-00394-1

**Published:** 2024-07-11

**Authors:** Torsten Bendl, Gina Maria Klein, Alexander Stöger

**Affiliations:** 1 Professur für Wissenschaftsgeschichte, Universität Regensburg, 93040 Regensburg, Deutschland; 2 Geschichte und Wissenschaftstheorie der Medizin, Universität Bielefeld, Postfach 100131, 33501 Bielefeld, Deutschland; 3 Cluster für Europaforschung | Nachwuchskolleg Europa, Universität des Saarlandes, Postfach 15 11 50, 66041 Saarbrücken, Deutschland

Erzählt man Teilnehmer:innen der GWMT-Tagung von den aktuellen Themen im Driburger Kreis, ist eine häufige Reaktion: „Ich erinnere mich! Ich war damals auch im Driburger Kreis, da habe ich noch studiert“ oder: „Ich weiß noch, wie ich dort als frisch Promovierende zum ersten Mal vorgetragen habe“. Fragt man weiter nach, so heißt es von fast allen Alters- und Berufsstufen: „Den Driburger Kreis gibt es schon ewig! Der ist entstanden, weil junge Wissenschaftler:innen keine Lust mehr hatten, ihren Professor:innen bei den Tagungen die Tasche hinterherzutragen.“ Erinnerungen von Momentaufnahmen des Driburger Kreises, die im Hinblick auf seine Geschichte seinen einzigartigen Charakter bestens ausdrücken.

Der Driburger Kreis, eine unverbindliche Gruppe von Akademiker:innen am Beginn ihrer Forschungskarriere (Early Careers) in der Wissenschafts‑, Medizin- und Technikgeschichte, die sich jedes Jahr im Vorfeld der Jahrestagung der Gesellschaft für Wissenschaften, Medizin und Technik (GWMT) trifft, ist eine Institution, aber nicht institutionalisiert. Als eine solche Assoziation wurde er 1962 gegründet, und dies betonen die Organisator:innen bis heute. Der Driburger Kreis ist eine wichtige Anlaufstelle für Studierende, Promovierende und Habilitierende, um ihre Forschungsthemen vorstellen und sich austauschen zu können. Er geht jedoch über seine Funktion als Anlaufstelle hinaus und verkörpert seit seiner Gründung unausgesprochen ein Mehr, das von Generation zu Generation weitergetragen wird – weil jede Generation es als essenziell betrachtet, ihre eigene Zukunft mitgestalten zu können.

## Von den Anfängen

Worin liegt das Geheimnis dieser beispiellosen, nicht institutionalisierten Institution der deutschen wissenschafts-, medizin- und technikhistorischen Landschaft? Womöglich liegt es in der Gründung aus der – wie der Mythos suggeriert – Frustration, den Professor:innen die Tasche hinterhertragen zu müssen. Um das zu klären, lohnt es sich, einen Blick auf die frühen Jahre des Driburger Kreises und seine Gründungsmitglieder zu werfen. Die historische Aufarbeitung stellt allerdings aufgrund der dünnen Quellenlage eine Herausforderung dar.

Die Erinnerungen von Fritz Krafft, einem der Gründungsmitglieder und späteren Professor für Wissenschaftsgeschichte in Hamburg und Marburg, Präsident der Gesellschaft für Wissenschaftsgeschichte und Begründer der Berichte der Wissenschaftsgeschichte, bieten aber doch einen besonderen Einblick in die Anfänge der Institution.

In einer Festschrift zum 65. Geburtstag von Günther Kerstein, einem Paten des Driburger Kreises, resümiert Krafft: „Eine Reihe von Instituten für Geschichte der Medizin und für Geschichte der Naturwissenschaften war kurz zuvor an den deutschen Universitäten neu eingerichtet oder in ihrem Stellenplan erweitert worden und schickte ihre Assistenten und älteren Studenten erstmals auf eine wissenschaftshistorische Tagung“ (Krafft 1969: 4). In Bad Driburg, so heißt es weiter, seien 1962 die unerfahrenen jungen Forscher:innen anfangs mit der unvertrauten Umgebung überfordert gewesen, hätten aber rasch zueinander gefunden und die Gelegenheit genutzt, um sich auszutauschen. Es mag keine explizite Erwähnung der Frustration als Taschenträger:in geben, wohl aber wurde betont, dass „wir […] uns von Anfang an gleichsam unter Gleichaltrigen mit dem Tagungs- und ihrem Wissenschaftsbetrieb kritisch auseinandersetzen [konnten] und […] uns darin einig [waren], daß wir vieles anders und einiges […] besser machen würden“ (Krafft 1969: 4). Mit „kritischen und weniger respektvollen Augen“ gewannen sie, die sie alle aus verschiedenen Fachrichtungen stammten, eine neue Perspektive auf die traditionsreiche Gesellschaft und ihre Tagungsaktivitäten und verspürten das Bedürfnis, sich darüber auszutauschen.

Im darauffolgenden Jahr fand die Jahrestagung der Deutschen Gesellschaft für Geschichte der Naturwissenschaften, Medizin und Technik (DGGNMT) in Schaffhausen statt. Die jungen Wissenschaftshistoriker:innen entschieden sich dazu, einen Tag vor Tagungsbeginn zusammenzukommen, um sich über selbstgewählte Themen auszutauschen, allerdings nicht in Schaffhausen, sondern in einer nahegelegenen kleineren Stadt – Bad Driburg. So kam der Driburger Kreis zu seinem Namen und zugleich zu seiner Nichtinstitutionalisierung. Denn der Driburger Kreis verstand sich, wie Krafft erläutert, von Anfang an als lose, verpflichtungsfreie Zusammenkunft als „Juniorenkreis“ (Krafft 1969: 6) der Wissenschafts‑, Medizin- und Technikgeschichte, die bewusst auf Einladungen, Mitgliedschaften oder eine formelle Verwaltung verzichtete. Diese Freiheit entsprach den Bedürfnissen der frühesten Mitglieder, sich zwanglos und unvoreingenommen austauschen zu können. Außerdem wollte man nicht den Verdacht erwecken, „die Gründung einer Gesellschaft in der Deutschen Gesellschaft oder einer zweiten neben ihr“ im Blick zu haben. Die einzige Formalität, die der Driburger Kreis in Kauf nahm, war die Ernennung eines „Sekretärs […], ohne daß der jeweilige Leiter allerdings diesen Titel […] trüge.“ Er war notwendig, um Reisestipendien von Dritten annehmen und verwalten zu können.

## Die Etablierung des Driburger Kreises

Fortan tagte der Driburger Kreis im Vorfeld der Jahrestagung der DGGMNT – anfangs noch in benachbarten Orten, in denen günstig Räume zur Verfügung standen, beispielsweise 1966 in Göttingen, als die Haupttagung in Braunschweig stattfand, oder 1968 im damaligen und mit Ernst Brüche (1900–1985) eng verbundenem „Haus der Physik“ in Mosbach mit dem Haupttagungsort Heilbronn in der Nähe (Krafft 1969: 8). Später wurden die beiden Tagungsorte zusammengelegt, wobei der Driburger Kreis an der Tradition festhielt, vor der Haupttagung stattzufinden. Und schon lange ist es üblich, dass die lokalen Organisator:innen der Haupttagungen den Driburger Kreis logistisch unterstützen, wenn es um Tagungsräume, Hotels und Pausenverpflegung geht.

Auch die Wahl der Tagungsthemen lag von Beginn an bei den Teilnehmer:innen der vorherigen Tagung. Von weitreichenden, praktischen Themen wie „Quellenkundliche und methodische Fragen“ (1964) oder „Neue Herausforderungen an die Geschichte der Naturwissenschaften“ (1999) über spezielle, den Geist der Zeit aufgreifende Themen wie „Entwicklung der instrumentalen Technik für die Forschung“ (1969) bis hin zu außergewöhnlichen Themen wie „Alchemie-Iatrochemie“ (1965) oder „Frösche“ (Stöger 2022) haben die Teilnehmer:innen des Driburger Kreises seit seinem Bestehen ein weites Spektrum ihrer Interessensgebiete ohne Vorbehalte ausfüllen können. Gelegentlich erwies sich die ungezwungene Vereinigung als besonders hilfreich, um Themen in den Blick zu nehmen, vor denen man in etablierteren Kreisen noch zurückschreckte. So befasste sich die DGGMNT erst 1995 auf Vorschlag des Driburger Kreises mit dem Thema der Geschlechterverhältnisse in Naturwissenschaften, Medizin und Technik, nachdem die Early-Career-Forscher:innen es bereits 1993 zu ihrem Rahmenthema gewählt hatten (Meinel & Renneberg 1996: 9 f.). Was nach außen wie eine unberechenbare Wildcard erscheinen mag und womöglich manche etabliertere Mitgliedern der wissenschaftshistorischen Gemeinschaft verunsicherte, ermöglichte letztlich neue Impulse und eine kritische Evaluation vorhandener Strukturen aus einer neuen Perspektive. Dieses Bestreben hat sich auch in späteren Generationen der Teilnehmer:innen fortgesetzt – so etwa, als Siegfried Bodenmann und Susan Splinter 2009 den Sammelband *Mythos – Helden – Symbole. Legitimation, Selbst- und Fremdwahrnehmung in der Geschichte der Naturwissenschaften, der Medizin und der Technik* herausgaben, basierend auf dem Tagungsthema des Driburger Kreises 2005 (Bodenmann & Splinter 2005, 2009: vii).

Die Teilnehmer:innen des Driburger Kreises waren und sind keine Fachfremden, sondern im Gegenteil jene, die als Teil der wissenschaftshistorischen Gemeinschaft ein besonders starkes Interesse an der Zukunft des Fachs haben, ohne durch Verpflichtungen gegenüber der Vergangenheit belastet zu sein. Befasst man sich mit den ersten Mitgliedern des Driburger Kreises, so fällt außerdem rasch auf, dass der Großteil von ihnen später seinen Weg in Professuren und zentrale Positionen innerhalb der wissenschaftshistorischen Fachcommunity fand. Da ist Brigitte Hoppe, die während der ersten Jahre im Driburger Kreis promoviert wurde und später die Professur für Wissenschaftsgeschichte in München erhielt. Oder Armin Hermann, ebenfalls während seiner Jahre im Driburger Kreis promovierend, der am Forschungsinstitut des Deutschen Museums in München arbeitete und schließlich den neugeschaffenen Lehrstuhl für Geschichte der Naturwissenschaften, Medizin und Technik in Stuttgart besetzte. Geschuldet der freien Struktur des Driburger Kreises lassen sich die Lebenswege vieler anderer Teilnehmer:innen über die Jahrzehnte dagegen nicht in ihrer Gänze erfassen. In den 62 Jahren des Bestehens des Driburger Kreises ist die Liste jener, die erst Teil dieser losen Zusammenkunft wurden und später in etablierten akademischen Positionen mit guten Erinnerungen an diese Zeit zurückdachten, nach wie vor im Wachsen. Und es ist wohl nicht übertrieben, wenn man annimmt, der Driburger Kreis habe an ihren Karrieren zumindest einen kleinen Anteil gehabt, indem er nicht nur den ungezwungenen Austausch über die eigenen Forschungsprojekte ermöglichte, sondern auch einen geschützten Raum für Gleichgesinnte schuf, die die Gelegenheit erhielten, sich nach ihren Bedürfnissen zu organisieren.

Bei aller Unabhängigkeit, die schon Fritz Krafft und seine Kolleg:innen betonten, war der Driburger Kreis aber von Beginn an mit der DGGMNT verbunden und auf ihre Hilfe angewiesen. So ermöglichten schon in den ersten Jahren Reisestipendien – anfangs von der Fritz-Thyssen-Stiftung, später aus Mitteln der GWMT bereitgestellt – überhaupt, dass sich die Studierenden und Promovierenden treffen konnten (Krafft 1969: 6). Auch stellten etablierte Wissenschaftshistoriker:innen Räumlichkeiten an ihren Instituten zur Verfügung, in denen sich der Driburger Kreis abseits der Jahrestagung der DGGMNT treffen konnte. Ein weiterer wichtiger Faktor war die enge Verständigung zwischen Vorstand und Organisator:innen. Sie sorgte dafür, dass diese Einrichtung als Institution der deutschen wissenschaftshistorischen Forschungsgemeinschaft ohne formellen Zwang Bestand haben konnte. Und nicht zuletzt die Ermunterung junger Forscher:innen und Student:innen durch ihre Dozent:innen und Professor:innen, am Driburger Kreis teilzunehmen und so den Einstieg in die wissenschaftshistorische Fachwelt zu finden, hat dazu beigetragen, dass über 60 Jahre und viele Generationen später der Driburger Kreis noch immer jährlich zusammenkommt.

## Gegenwart und Zukunft – eine Institution ohne Institutionalisierung

Auch heute noch treffen sich Early-Career-Wissenschafts‑, Medizin- und Technikhistoriker:innen im Vorfeld der Jahrestagung der GWMT, um ihre Forschungsprojekte vorzustellen, über wissenschaftshistorische Themen zu diskutieren und sich auszutauschen. Nach wie vor gibt es keine festen Strukturen, Mitgliedschaften oder gar eine Satzung. Tagungsthemen und Organisator:innen werden von den Anwesenden gewählt, die Tagungsorte sind inzwischen dieselben wie die der GWMT-Tagung. Ebenso hat sich die Unterstützung durch die GWMT und die lokalen Organisator:innen erhalten, die erst ermöglichen, dass sich der Driburger Kreis regelmäßig treffen kann. Seit 1999 werden die Calls auf elektronischem Wege verteilt, wie beispielsweise über H‑Soz-Kult (Broemer 1999), und seit 2022 besitzt der Driburger Kreis eine Webseite (https://driburgerkreis.de) und eine Social-Media-Präsenz (@DriburgerKreis). Er nutzt digitale Möglichkeiten für zusätzliche Angebote. Inzwischen besteht das Organisationsteam aus drei Early Careers, um idealerweise die Themenfelder der Wissenschafts‑, Medizin- und Technikgeschichte sowie verschiedene Karrierestufen im wissenschaftlichen und wissenschaftsnahen Betrieb abzudecken. Außerdem kann so die Arbeitslast der Freiwilligen angesichts prekärer Beschäftigungssituationen besser verteilt werden. Die Organisation hat sich modernisiert, aber nicht verändert, und das nicht etwa, weil der Driburger Kreis eine dezidierte Traditionslinie zu bewahren sucht – im Gegenteil.

Jedes Organisationsteam entdeckt sich neu, ohne andere Pflichten oder Anforderungen vorheriger Generationen als die jährliche Zusammenkunft interessierter Early-Career-Forscher:innen zu ermöglichen. Dennoch ist das hier keine Fortschritts- oder Umbruchsgeschichte, obwohl die letzten 62 Jahre zweifellos genug Fortschritt und Umbruch gesehen haben. Jede Generation an Teilnehmer:innen und Organisator:innen des Driburger Kreises sieht aufs Neue dieselbe Notwendigkeit darin, einen Raum zu schaffen, in dem sich Early Careers austauschen, erste Erfahrungen sammeln und etablierte Strukturen kritisch evaluieren können. Als solches ist der Driburger Kreis eine wichtige Institution der deutschsprachigen wissenschaftshistorischen Fachgemeinschaft – nicht nur als Möglichkeit für zukünftige Akademiker:innen, erste Tagungserfahrungen zu sammeln und Gleichgesinnte kennenzulernen, sondern vor allem als ein Format, das es erlaubt, immer wieder aufs Neue auf die aktuellen Bedürfnisse seiner Teilnehmer:innen einzugehen und ihnen die Chance zu geben, sich selbst zu organisieren.

Als solche Institution hat der Driburger Kreis auch nach 62 Jahren weder an Wichtigkeit noch an der Zahl Interessierter eingebüßt. Während der Pandemie, als der persönliche Austausch sich als besonders schwierig erwies, boten die online abgehaltenen Tagungen gerade jenen eine erste Möglichkeit, externes Feedback zu ihrer Arbeit zu erhalten, die ihre Projekte kurz vor oder während des Lockdowns begonnen hatten. In Zeiten zunehmend prekärer Beschäftigungsverhältnisse an Universitäten und anderen Forschungs- und Bildungseinrichtungen und damit verbundenen zusätzlichen Belastungen bietet der Driburger Kreis neben Workshops auch den wichtigen informellen Erfahrungsaustausch zwischen den Teilnehmer:innen. Bei den Treffen haben sie die Möglichkeit, Fragen zu stellen und auf Angebote aufmerksam zu werden, die bei den kaum zu durchdringenden Strukturen und komplexen Erwartungen ohne Hinweise und Hilfestellung praktisch nicht zu bewältigen sind. Darüber hinaus können Early Careers dort mit Mitgliedern anderer wichtiger Gruppierungen und Organisationen ins Gespräch kommen und sich so innerhalb und außerhalb der wissenschaftshistorischen Fachgemeinschaft weiter engagieren.

Der Driburger Kreis illustriert die essenzielle Rolle informeller Netzwerke in der akademischen Welt. Obwohl nur durch seine ungewöhnliche Form der assoziierten Unabhängigkeit erst möglich, ist er weit mehr als eine zwanglose Zusammenkunft von Wissenschaftshistoriker:innen. Er verkörpert eine lebendige Tradition des kritischen Denkens und der kollektiven Unterstützung, die die Wissenschaftsgemeinschaft über Generationen hinweg bereichert hat. In einer sich ständig verändernden wissenschaftlichen Landschaft bleibt der Driburger Kreis eine Institution ohne Institutionalisierung: eine wesentliche Plattform für Early-Career-Wissenschaftshistoriker:innen, um sich zu vernetzen, zu wachsen und die Zukunft ihres Fachs mitzugestalten.
